# Models of Trigeminal Activation: Is There an Animal Model of Migraine?

**DOI:** 10.3390/brainsci14040317

**Published:** 2024-03-27

**Authors:** Eleonóra Spekker, Annamária Fejes-Szabó, Gábor Nagy-Grócz

**Affiliations:** 1 Interdisciplinary Research Development and Innovation, Center of Excellence, University of Szeged, H-6725 Szeged, Hungary; 2 HUN-REN–SZTE Neuroscience Research Group, University of Szeged, H-6725 Szeged, Hungary; fejesannamaria@yahoo.co.uk; 3 Department of Theoretical Health Sciences and Health Management, Faculty of Health Sciences and Social Studies, University of Szeged, Temesvári Krt. 31., H-6726 Szeged, Hungary; nagy-grocz.gabor@szte.hu; 4 Preventive Health Sciences Research Group, Incubation Competence Centre of the Centre of Excellence for Interdisciplinary Research, Development and Innovation of the University of Szeged, H-6720 Szeged, Hungary

**Keywords:** trigeminal system, trigeminal activation, migraine, primary headache, animal model, dura mater, trigeminal ganglion, FHM, nitroglycerin

## Abstract

Migraine, recognized as a severe headache disorder, is widely prevalent, significantly impacting the quality of life for those affected. This article aims to provide a comprehensive review of the application of animal model technologies in unraveling the pathomechanism of migraine and developing more effective therapies. It introduces a variety of animal experimental models used in migraine research, emphasizing their versatility and importance in simulating various aspects of the condition. It details the benefits arising from the utilization of these models, emphasizing their role in elucidating pain mechanisms, clarifying trigeminal activation, as well as replicating migraine symptoms and histological changes. In addition, the article consciously acknowledges the inherent limitations and challenges associated with the application of animal experimental models. Recognizing these constraints is a fundamental step toward fine-tuning and optimizing the models for a more accurate reflection of and translatability to the human environment. Overall, a detailed and comprehensive understanding of migraine animal models is crucial for navigating the complexity of the disease. These findings not only provide a deeper insight into the multifaceted nature of migraine but also serve as a foundation for developing effective therapeutic strategies that specifically address the unique challenges arising from migraine pathology.

## 1. Introduction

Migraine, as a neurological disorder, affects millions of people worldwide, imposing a significant burden on individuals and society alike. The condition is intricately linked to trigeminal activation, a key component of the pain-sensing system. Animal experimental models play a critical role in providing insights into the functioning of migraine and the trigeminal system. Through these models, various aspects of the disease’s pathomechanism can be examined, contributing to more precise diagnostics and the development of more effective therapies. This review offers an overview of the fundamental characteristics of migraine and trigeminal activation, the crucial role of animal experimental models, and their significance in advancing clinical treatments and therapeutic strategies for the condition.

## 2. The Multifaceted World of Migraine: Characteristics, Pathomechanisms, Associated Disorders, and Therapeutic Challenges

Migraine is a primary, episodic headache disorder distinguished by diverse manifestations of neurological, gastrointestinal, and autonomic alterations [1]. The ongoing Global Burden of Diseases, Injuries, and Risk Factors Study consistently recognizes migraine as a prominent contributor to global disability, especially among individuals under the age of 50 [2,3]. Moreover, conditions frequently occurring alongside migraine, such as neck pain, depression, and anxiety, rank among the top ten causes of global disability [4,5].

Migraine attacks are often preceded by warning signs (such as fatigue, euphoria, irritability, food cravings, or constipation) [6,7] and the phenomenon of aura [8], which is believed to originate in the hypothalamus, brainstem, and cortical areas [9,10]. The attacks are accompanied by severe, throbbing headaches typically affecting one side and intensifying with increased intracranial pressure, and their duration varies but generally lasts between 4–72 h. Migraine headaches often come with nausea and potential vomiting. Patients are frequently sensitive to light and sound during the attacks [10,11]. Furthermore, it may be accompanied by abnormal skin sensitivity (allodynia), muscle tenderness, and osmophobia [12]. Exacerbation of headache due to routine physical activity is an extremely sensitive characteristic of migraine headaches, occurring in over 95% of sufferers [13]. The symptoms observed from the prodromal stage to the headache phase of migraine indicate the dysfunction of several neuronal systems. According to The International Classification of Headache Disorders-3 beta (ICHD-3) criteria, the vestibular symptoms of migraine, such as vertigo and dizziness, are now identified with the diagnosis of migraine with brainstem aura, emphasizing that these symptoms likely reflect changes in neural activity in the brainstem and vestibular system rather than alterations in blood flow through the basilar artery [14].

Migraine is a complex headache condition that can be classified into different types. Episodic migraine (EM) implies that the patient experiences migraine attacks periodically, typically for up to 14 days a month. In contrast, chronic migraine (CM) involves more frequent headaches, occurring for more than 15 days a month [15]. EM can evolve into CM when the frequency of headaches gradually increases, leading to a persistent migrainous state [16]. Factors influencing progression include older age, female gender, Caucasian ethnicity, low educational level, low socioeconomic status, and genetics. Obese individuals and snorers are two to five times more likely to develop CM. Additional risk factors include head or neck injury, comorbid depression, stressful life events, asthma, and allergic rhinitis. Factors promoting remission include lower baseline headache frequency and the absence of cutaneous allodynia, especially thermal [17]. The regular and excessive use of migraine medications can result in medication-overuse headache (MOH). The ICHD-3 beta criteria conceptualize medication overuse as a behavior rather than being based on causality. MOH is characterized by a patient using triptans, ergot alkaloids, mixed analgesics, or opioids for ≥10 days per month; simple analgesics or nonsteroidal anti-inflammatory drugs (NSAIDs) for ≥15 days per month; or the concurrent use of multiple substances for more than 10 days per month [14].

**Figure 1:**
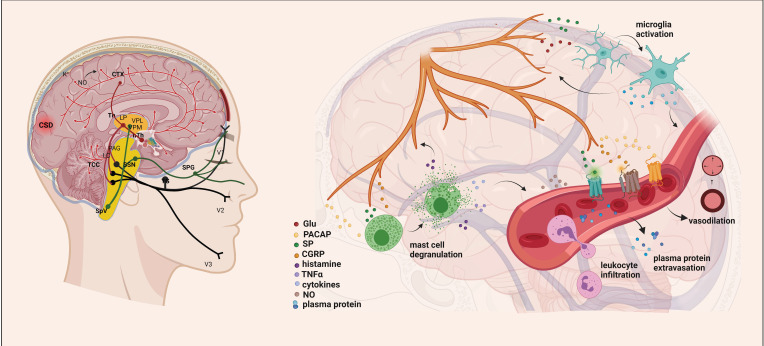
Exploring the Mechanisms Underlying Migraine Attacks [23]. During migraines, various brain regions are affected, including the dorsolateral pons and dorsal midbrain: NRM, DR, LC, and PAG. These nuclei influence the activity of the trigeminocervical complex and play a role in pain transmission. The initiation and spread of migraine attacks are determined by significant increases in extracellular K^+^, NO, and glutamate concentrations. Cortical spreading depression (CSD) can activate sensory neurons in the trigeminal ganglion, releasing molecules like ATP, glutamate, K^+^, H^+^, AA, and NO locally, leading to their diffusion and activation of meningeal nociceptive neurons. This results in a localized increase in neuroactive inflammatory mediators and sensitization of brainstem regions relevant to pain. Stimulation of the trigeminal nerve causes the release of neuropeptides, initiating neurogenic inflammation with four main features: vasodilation, increased vascular permeability, leukocyte infiltration, and activation of glial cells, along with mast cell degranulation, leading to increased production of inflammatory mediators such as cytokines and chemokines. AA, arachidonic acid; CTX, cortex; NO, nitric oxide; CSD, cortical spreading depression; Th, thalamus; hTh, hypothalamus; LP, lateral posterior nucleus; VPM, ventral posteromedial nucleus; VPL, ventral posterolateral nucleus; PAG, periaqueductal grey matter; LC, locus coeruleus; TCC, trigeminocervical complex; SSN, superior salivatory nucleus; SpV, spinal trigeminal nucleus caudalis; TG, trigeminal ganglion; SPG, sphenopalatine ganglion; V1, ophthalmic nerve; V2, maxillary nerve; V3, mandibular nerve; Glu, glutamate; CGRP, calcitonin gene-related peptide; SP, substance P; PACAP, pituitary adenylate cyclase-activating polypeptide; TNFα, tumor necrosis factor alpha; NRM, nucleus raphe magnus; DR, nucleus raphe dorsalis.

Despite extensive investigation, our understanding of the pathogenesis of migraine disease remains limited. Several key factors, including vascular dysfunction, cortical spreading depression (CSD), trigeminovascular pathway activation, and inflammatory and oxidative conditions, have been implicated as fundamental components contributing to the development of migraine pain [18]. Several neurochemical changes play a role in the development of migraine, and one key element is calcitonin gene-related peptide (CGRP) and pituitary adenylate cyclase-activating polypeptide (PACAP). These peptide molecules are involved in triggering and sustaining headaches [19]. Neuropeptide release can lead to mast cell degranulation and plasma extravasation, initiating neurogenic inflammation (NI) [20]. Simultaneously, second-order neurons are activated in the caudal trigeminal nucleus (TNC), and their axons ascend to terminate in the thalamus, transmitting nociceptive information to the primary somatosensory cortex. Neuroimaging studies have identified additional regions of the central nervous system (CNS), such as the cerebellum, insula, and pulvinar, that may play a role in modulating the sensation of pain [21,22] (Figure 1).

Functional and structural changes occurring in various areas of the brain may contribute to the development of migraines. Various imaging techniques comparing migraineurs to control subjects reveal increased activation in specific areas, including the periaqueductal grey, red nucleus, substantia nigra, hypothalamus, posterior thalamus, cerebellum, insula, cingulate and prefrontal cortices, anterior temporal pole, and hippocampus. Conversely, diminished activation is observed in the somatosensory cortex, nucleus cuneiformis, caudate, putamen, and pallidum [24,25,26,27,28]. Among the structural changes are thickening of the somatosensory cortex, increased gray matter density in the caudate, and gray matter volume loss in various areas such as the superior temporal gyrus, inferior frontal gyrus, precentral gyrus, anterior cingulate cortex, amygdala, parietal operculum, middle and inferior frontal gyrus, inferior frontal gyrus, and bilateral insula. These changes may depend on the frequency of migraine attacks and other chronic pain conditions [29,30,31,32,33].

The genetic background of migraine is complex, and the role of genetic factors in the development of the condition is still partially unexplored. Family occurrences indicating the frequency of migraine attacks in certain families and the study of risks associated with specific gene variants suggest that genetics may play an important role in migraine predisposition. Family history suggests a genetic predisposition to migraine, initially observed in patients with familial hemiplegic migraine (FHM). Three genes linked to FHM regulate glutamate availability in the synapse, affecting neurotransmission and synaptic plasticity [34,35,36]. Large-scale genome-wide association studies have identified 13 susceptibility gene variants for migraine, with three influencing glutaminergic neurotransmission and two related to synaptic development and plasticity [37,38,39,40]. These findings provide insights into the generalized neuronal hyperexcitability observed in the migraine brain. However, it is important to note that genetic predisposition is just one factor in the development of migraines.

Several triggering factors are known, such as certain foods, stress, weather changes, hormonal influences, and certain habits like excessive caffeine consumption or lack of sleep [41,42,43,44,45].

The intricacy of migraines has hindered the comprehensive modeling of this disorder in animals, presenting a significant challenge that still needs to be addressed. With the appropriate diagnosis and treatment, the quality of life for those affected and the manageability of their condition can be significantly improved.

## 3. Exploring Animal Models in Migraine Research: Unraveling Complexity

Utilizing animal models to study human diseases has been immensely helpful, contributing to the enhanced understanding of brain disorders and the development of innovative therapeutic approaches. Various methods are usually employed in animal experimental research to study the role of age, such as comparing young and old animals, analyzing age-related changes in the frequency and severity of the disease, or investigating age-related biological processes. Although the subjective nature of migraine headache limits detailed investigations in animals, we would emphasize that such models remain valuable in understanding fundamental mechanisms. Behavioral and physiological markers are often employed in animal experimental studies to assess headache. These markers may include behavioral signs related to pain, such as head scratching or restlessness, as well as autonomic nervous system responses, such as changes in heart rate or elevation in stress hormone levels. Such markers can aid in estimating the degree and intensity of headache in the animal experimental environment. Although alternative methods such as in vitro models or computer simulations as well as advanced AI-based techniques are playing an increasingly significant role, they may not always be as effective in examining complex disease mechanisms as animal experiments.

**Figure 2:**
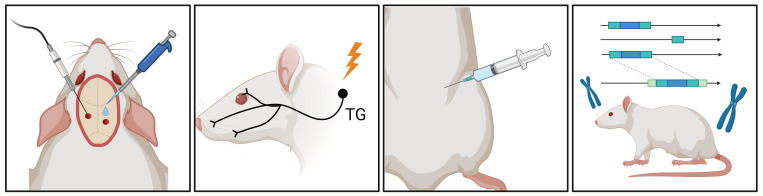
Migraine Research Insights: Schematic Overview of Animal Models. In migraine research, various animal models are available, including electrical or chemical stimulation of the dura mater, electrical stimulation of the trigeminal ganglion, systemic administration of nitroglycerin (NTG), and the utilization of various genetic models. These models facilitate the study of diverse migraine-triggering mechanisms and pathophysiological processes in animal experiments. TG, trigeminal ganglion.

Our understanding of migraine pathophysiology in recent decades is largely based on animal experiments. These experiments explore the nociceptive pathways of the trigeminovascular system and their ascending projections to the brainstem and diencephalic nuclei as well as the control these structures exert over nociceptive and other sensory processing pathways that lead to migraine symptoms. The existing migraine models, like models in other scientific fields, are essentially simplified representations of reality due to the constraints of available knowledge. Advancements in current models, alongside human research, have the potential to increase the translational efficacy of headache models. The expanding use of genetically modified animals and innovative methods may result in the development of novel, distinctive models (Figure 2).

### 3.1. Cranial Stimulation Models: Investigating Migraine Mechanisms via Electrical Activation

The experimental model of migraine involves the electrical stimulation of the Gasserian ganglion, resulting in plasma extravasation within the dura mater [46,47]. The greatest advantage of electrical stimulation is that specifying stimulation parameters (current intensity: mA; voltage: V; pulse duration: ms–min; frequency: Hz) provides a controllable, reliable, monitorable, and repeatable method in contrast to mechanical, chemical, or magnetic stimulation. Furthermore, the use of electrical electrodes allows for precise and accurate stimulation [48]. Based on current research findings, electrical stimulation of the Gasserian ganglion leads to modifications in the distribution of CGRP content, impacting both the peripheral and central processes of primary sensory neurons. It is suggested that the decline in CGRP immunoreactivity indicates a depletion of this neuropeptide from the central terminals [49,50].

Under experimental conditions in one study, electrical stimulation of the trigeminal ganglion triggered plasma protein extravasation in the dura mater. However, stimulation of the trigeminal ganglion did not result in uniform brainstem activation in areas related to migraine, although it activated the pain modulatory system [51]. Additionally, this model allowed for the identification of a direct correlation between PACAP (another vasoactive neuropeptide) and the kynurenine pathway (KP) during trigeminal vascular system activation [52]. In other studies, repeated stimulation of the trigeminal ganglion led to allodynia, and it was demonstrated that the effect of sumatriptan in this model is primarily mediated through 5-hydroxytryptamine 1B/1D (5-HT_1B/D_) receptors [53]. It is important to note that a drawback of this model is the induction of inflammatory responses due to the insertion of electrodes into the brain parenchyma [54].

Stimulation of meningeal nerve terminals innervating the superior sagittal sinus (SSS) through electrical stimulation as well as the transverse sinus or middle meningeal arteries [55,56,57] serves as a preclinical model for studying migraines. The model has been employed to investigate the central effects of drugs such as ergotamine, sumatriptan, and acetylsalicylic acid, which inhibit the transmission of trigeminal nociceptive information in the brainstem. SSS stimulation allows for the analysis of c-Fos protein expression, a crucial marker of neuronal activity, revealing, for example, the role of glutamate in neurotransmission within the trigeminocervical complex [58,59,60,61]. Based on these findings, the SSS stimulation model proves to be a valuable tool for understanding migraine mechanisms and developing potential treatment strategies.

Electrical stimulation of the dura mater in rats, activating thin myelinated and unmyelinated nerve fibers, increases meningeal blood flow. This response can be diminished by 5-HT1 receptor agonists and eliminated by a CGRP receptor antagonist [62,63,64].

### 3.2. Migraine Models: Chemical Stimulation of the Dura Mater

One of the migraine models used in animal experiments involves the chemical stimulation of the dura mater. In this method, various irritants and inflammatory mediators (capsaicin, complete Freund’s adjuvant (CFA), and inflammatory soup (IS)) are applied to the dura mater. The application or infusion of inflammatory or algesic substances onto the dura mater or chemical stimulation of the dural receptive fields in rats induces heightened sensitivity to mechanical and thermal stimulation, accompanied by the direct activation of the trigeminal ganglion [65,66,67,68,69].

In animal experiments, capsaicin is used through either direct application to the exposed dura mater in a rat open cranial window preparation or intracisternal injection. This leads to the activation of trigeminovascular nociceptive afferents [70,71]. Triggering trigeminal nociceptive nerve fibers with capsaicin induces nociceptive behavior associated with face grooming [72,73,74]. Additionally, it brings about changes in blood flow within meningeal tissues and induces neurochemical alterations in second-order neurons of the trigeminal brainstem nuclei. Models utilizing capsaicin application are widely acknowledged as effective representations of meningeal nociception and headache [71].

Edvinsson et al. proposed that the persistent trigeminal activation caused by administering dural or temporomandibular CFA could serve as a model for the shift from episodic to chronic migraine [75]. Nociceptor activation leads to the release of CGRP and other neural mediators, initiating a local inflammatory response. This process likely triggers ongoing activation and sensitization of the trigeminal system, establishing a self-reinforcing mechanism [76,77,78]. This effect is comparable to the consequences of recurrent migraine attacks, potentially giving rise to continuous neurogenic inflammation, also known as neurogenic neuroinflammation [72]. Applying CFA to the dura or temporomandibular region may induce sustained activation in the trigeminal system, contributing to the onset of chronic migraine [68].

Oshinsky and Gomonchareonsiri administered IS treatment three times a week for up to four weeks, revealing that repeated IS infusions over weeks resulted in a sustained reduction in periorbital pressure thresholds [79]. A study by Lukács et al. revealed that CFA or IS application onto the dura induces changes in pERK1/2, IL-1β, and CGRP-positive nerve fibers in the TG [67]. Laborc et al. used IS or CFA topically on the dura, finding IS induced short-term c-Fos activation, while CFA showed no significant difference in c-Fos-positive cells [68]. Spekker et al. noted that IS induces sterile neurogenic inflammation in the dura mater, leading to an increase in the area covered by CGRP and transient receptor potential vanilloid type 1 (TRPV1) immunoreactive fibers as well as a rise in the count of neuronal nitric oxide synthase (nNOS)-positive cells in the TNC, which was modulated by sumatriptan, and kynurenic acid (KYNA) [80]. In another study by Spekker et al., behavioral results indicated that IS infusion enhances nociceptive responses. They found that the multiple administrations of IS on the dura mater can cause a significant decrease in mechanical pain thresholds in both the orofacial von Frey test and the hind-paw mechanical allodynia test. Furthermore, they observed increased facial grooming and scratching behavior [81]. Wieseler et al. reported IS administration leading to increased IL-1β, TNFα, and CD11b levels in TNC. IS also impacts animal behavior, inducing persistent changes in periorbital pressure thresholds, altered locomotor activity, and specific grooming behaviors [82]. Melo-Carrillo and colleagues noted increased resting and freezing behavior [83], while Malick et al. demonstrated reduced appetite in rats subjected to simultaneous chemical and mechanical dural stimulation [84]. In a large animal model, recurrent IS stimulation of the dura decreased locomotor behavior and induced pain-related behaviors, making it a relevant acute migraine animal model [85].

### 3.3. Exploring the Migraine-Inducing Properties of Nitroglycerin: Mechanisms, Neurological Impacts, and Experimental Models

Nitroglycerin (NTG) is a medication commonly used to treat angina by dilating blood vessels, reducing the workload on the heart, and improving blood flow. NTG was initially deployed in 1879 to address angina, and ever since, it has been integral to the management of relieving chest pain associated with angina [86]. Nevertheless, its ability to dilate blood vessels may result in headaches for certain individuals [87]. Moreover, it has been noted that among migraine sufferers, a form of seizure without aura can manifest hours after the headache subsides [88]. The effect of NTG has been extensively studied and thoroughly documented in animal migraine models, making it of fundamental importance in migraine research. Its ability to reliably induce migraine-like symptoms in animal models allows researchers to closely mimic the physiological and symptomatic aspects of migraine attacks, facilitating the investigation of underlying mechanisms and the development of potential therapeutic interventions.

NTG is a highly permeable and lipophilic organic nitrate, and its ability to donate nitric oxide (NO) is believed to be the primary mechanism underlying its migraine-inducing effects [89]. Initially, it was believed that the conversion of NTG to NO primarily occurred within vascular walls, influencing vascular tone exclusively. However, the discovery of NO’s presence in various tissues revealed its significant modulatory roles in the nervous system and inflammatory responses [90]. Due to its remarkable permeability and diffusion properties, NO acts as a pivotal neurotransmitter in the CNS, affecting nearby neural and glial structures [90].

Endogenously, the production of NO involves three distinct isoforms of NO synthase (NOS), which form dimers and catalyze the oxidation of l-arginine into l-citrulline. These isoforms, namely nNOS, inducible NOS (iNOS), and endothelial NOS (eNOS), were initially named based on the tissues in which they were identified but are extensively distributed throughout both the peripheral and CNS [91]. In the endothelium, eNOS produces NO in response to various stimuli, including shear stress from blood flow [92]. NO diffuses to nearby vascular smooth muscle cells, where it activates the enzyme soluble guanylyl cyclase (sGC), leading to increased levels of cyclic guanosine monophosphate (cGMP). Elevated cGMP levels cause relaxation of smooth muscle cells, leading to vasodilation and increased blood flow. This process helps regulate blood pressure and maintain cardiovascular health [92]. In the nervous system, NO acts as a neurotransmitter and neuromodulator. In neurons, nNOS synthesizes NO, which can diffuse across cell membranes and modulate the activity of neighboring cells. NO is involved in various neuronal functions, including synaptic plasticity, learning, and memory [93]. It also plays a role in regulating the release of other neurotransmitters such as glutamate and dopamine [94]. Beyond these, NO has antimicrobial properties and is involved in the immune response to pathogens. Macrophages and other immune cells express iNOS in response to inflammatory signals, leading to the production of large amounts of NO [95]. High levels of NO can help kill bacteria, viruses, and other pathogens by damaging their DNA or disrupting their metabolic processes [94]. However, excessive NO production can also contribute to tissue damage and inflammation in conditions such as septic shock [96]. Overall, NO is a versatile signaling molecule involved in numerous physiological processes in the human body, including cardiovascular regulation, neurotransmission, immune response, and smooth muscle function. Dysregulation of NO signaling is implicated in various diseases, including hypertension, atherosclerosis, neurodegenerative disorders, and erectile dysfunction.

The connection between NO, NTG, and headache has been recognized for a long time. Intravenous administration of NTG induces a mild-to-moderate bifrontal, throbbing headache within minutes, affecting both migraineurs and non-migraineurs, thus serving as a reliable model of vascular headache [97]. This headache is linked to NTG’s potent vasodilatory effects, believed to be mediated by its conversion into NO within the endothelial layer of the vascular wall [98,99]. However, while NTG can provoke a mild-to-moderate headache in non-migraineurs, it can trigger a headache attack resembling migraines exclusively in migraineurs [100,101] or individuals with a family history of migraines [102]. This migraine-like headache may onset within 45 min of NTG administration or be delayed by up to 4 to 5 h after the initial moderate headache subsides. Crucially, NTG also triggers the occurrence of common premonitory and associated symptoms [101]. The temporal pattern and clinical characteristics of these NTG-mediated migraine attacks suggest a downstream mechanism of action within the CNS. NTG has the ability to trigger the activation and sensitization in the trigeminal system of humans, a phenomenon observed in migraineurs, as well. Moreover, it stimulates various anatomical regions implicated in migraines, including the cervical spinal cord area, trigeminal nuclei, brainstem, and hypothalamus [9,103]. Migraine attack induced by NTG also increase levels of CGRP [104], a significant factor in migraine pathology [105,106]. Moreover, antimigraine agents like sumatriptan have been shown to diminish NTG-induced migraine attacks [107], thereby validating the model’s effectiveness.

On the other hand, numerous experiments, primarily conducted in rodents, have demonstrated that NTG can activate and sensitize the anatomical structures associated with migraines. The use of NTG in animal models is known to be both acute and chronic. Systemic administration of NTG as an acute model is able to increase the levels of c-Fos in rats [108], which indicates the activation of trigeminal system in many areas that correlate with migraine. Furthermore, NTG has also been shown to influence nNOS [76], CGRP [109], calcium/calmodulin-dependent protein kinase II alfa (CAMKIIα) [110], TRPV1 [111], nuclear factor kappa B (NF-κB) [112], and cyclooxygenase-2 (COX-2) [113] levels in the trigeminal system of rats. In addition to this, 5-HT and its transporter levels increased in the trigeminal system of rats after NTG administration [109,114]. On the other hand, administration of NTG led to a notable reduction in the ambulation distance observed in rats [115], which can be compared to the loss of movement seen in migraineurs. The use of NTG in animal studies can be justified by the fact that changes observed in human migraine patients have also been described in animals after NTG administration. An example of this observation is the involvement of the KP (kynurenine pathway) in migraine. KYNA, a crucial molecule in the KP, functions as an endogenous antagonist of N-methyl-D-aspartate (NMDA) receptors [116,117]. Within glutamate receptors, NMDA receptors have a central role in the pathomechanism of migraine [118]. Curto and her colleagues described abnormalities of various metabolites of the KP in the serum of patients with cluster headache and chronic migraine [119,120]. Similar alterations to these changes have been identified in rats, where the protein levels of several KP enzymes decreased four hours after systemic NTG administration [121]. In essence, NTG can induce alterations akin to those observed in human migraineurs.

In addition to the acute model of NTG, a chronic model is also known, which aims to investigate chronic migraine. By employing chronic intermittent administration of NTG, Pradhan and her colleagues devised a testing method that mimics the transition of migraine from an acute phase to a chronic condition [122]. In this chronic model, administering NTG every other day for nine days resulted in two different pain conditions: immediate hyperalgesia after each NTG injection and gradually increasing basal hypersensitivity [123]. Numerous animal studies support the possibility of repeated administration of NTG to induce activation and sensitization of the trigeminal system [123]. In addition to this, it has been described that chronic administration of NTG can trigger phenomena that are correlated to migraine, such as periorbital and perimasseter mechanical hyperalgesia [79,124], reduced locomotor activity [125], photophobia [126], and facial expressions of pain [125]. Besides these data, chronic administration of NTG can increase the CGRP and PACAP plasma levels in rats [127], suggesting the reliability of the model. PACAP, a member of the vasoactive intestinal peptide (VIP) family, has been shown to play a role in migraine [128].

Certainly, the NTG model possesses certain aspects that could be viewed unfavorably. It is crucial to emphasize that NTG does not induce migraine attacks in every individual; hence, care should be exercised when employing it in animal research. In the context of animal studies, it is worth mentioning that the commonly utilized dose of 5–10 mg/kg is significantly higher than the dosage administered in humans by orders of magnitude.

While NTG-induced models provide valuable insights, it is essential to recognize that migraine is a complex and multifactorial condition, and NTG may not fully replicate all aspects of migraine pathology. Findings from this experimental model can contribute to our understanding of migraine and potentially inform the development of new therapeutic approaches.

### 3.4. The Role of Genetic Factors in the Development of Migraine: Models and Research Approaches

Migraine shows a strong familial clustering, which is more pronounced in migraine with aura than in migraine without aura [129]. This allows us to conclude that genetic factors also play a role in the development of the disease, at least partially interacting with environmental factors. Many studies have been performed to search for the genetic background, such as genome-wide association studies (GWAS) and polygenic risk score (PRS) studies [130], which have identified many migraine-predisposing genes and confirmed that, from a genetic point of view, migraine is basically a disease with polygenic inheritance, which is caused by interaction between genetic and environmental factors.

At the same time, it is known that there are very rare but proven monogenic versions of migraine in which a single gene mutation can cause the disease. These have already given an opportunity to develop genetically modified animal models for migraine, which help better the understanding of the pathomechanism of migraine [131].

Among migraines with monogenic inheritance, the best known is the autosomal dominant FHM. Four subtypes are distinguished based on that in which gene the mutation exists: FHM1, FHM2, FHM3, and FHM4.

The FHM1 subtype includes migraineurs who carry a mutation affecting the CACNA1A gene located on chromosome 19p13.13 [132]. This gene encodes the alpha1 A subunit of the so-called CaV2.1 voltage-gated calcium channel, which plays, among others, an important role in the regulation of neurotransmitter release in neuronal synaptic nerve endings [133]. Several different mutations of this gene have been identified. The common feature of the mutations causing FHM1 is that they increase the function of the channel and therefore increase the influx of calcium into the cells, which leads to increased glutamate release and neuronal excitability in humans [134] and in mice, too [135]; these are so-called gain-of-function mutations.

There are transgenic mouse strains only for a few mutations among the many mutations affecting the CACNA1A gene identified in humans. The two knock-in mouse models are the mice with the milder R192Q and mice with the severer S218L missense mutation, which have similar visible symptoms as patients suffering from FHM1 carrying the same mutation [132,133,134,135,136,137,138]. These mice exhibit several differences compared to wild-type mice that can be paralleled with the known pathomechanism of migraine. Pain sensation is partially altered; i.e., the baseline facial pain is increased as measured by mouse grimace scale [139]. Transgenic animals exhibited photophobia-like behavior and several symptoms indicating spontaneous head pain induced by restraint and novelty stress. These behaviors were ipsilateral, had increased frequency, and showed sexual dimorphism and recovered dose-dependently by painkillers [140]. Interestingly, reactions to noxious thermal, mechanical, and chemical stimuli were unchanged in these animals [140]. Facilitated depolarization-evoked CGRP release from the trigeminal ganglion can be shown [130]. Moreover, there are some data suggesting the role of altered purinergic signaling induced by CGRP in changed pain-related molecular composition and facilitated signal transmission of pain in the trigeminal system [141]. In vitro and in vivo studies suggest that CSD susceptibility and underlying mechanisms such as release of excitatory neurotransmitters, synaptic transmission mediated by glutamatergic neurons, neuronal calcium concentrations, and the synaptic morphology are altered [142,143,144,145]. Changes related to synaptic plasticity can also be observed at the synaptic level in the cerebral cortex, brainstem, cerebellum, and hippocampus in in vitro and/or in vivo studies [136,142,143,146,147,148,149]. These animals showed changes in a number of substances that play an important role in the inflammatory process at protein and gene expression levels without triggering CSD and after CSD, which suggests that they are more susceptible to neuroinflammation [150,151,152]. Moreover, in the plasma of mutant mice, a changed concentration of some metabolites can be observed, which can be linked to compensatory mechanisms after excitation induced by CSD compared to wild-type animals [153]. At the cortical and subcortical level, CSD also induced alteration in the concentration of numerous metabolites and peptides [154]. The results on learning and memory are conflicting because learning and memory were impaired as measured by Morris in water maze and fear-conditioning experiments and a novel object recognition test, but increase of excitatory transmission and long-term potentiation (LTP) were measured in the hippocampus [149]. In relation to stress-induced migraine attacks, the restraint of stress enhanced the concentration of corticosterone in plasma of mice but did not affect CSD susceptibility, while corticosterone administered subcutaneously increased the frequency of CSD [155]. In line with the disrupted sleep observable in migraineurs, mutant mice have changed behavior related to sleep–wake rhythm, adapting faster to the light–dark cycle phase shifts and showing enhanced waking patterns in the active dark period after a 6 h sleep deprivation [156]. Differences related to sex can also be detected in mutant mice, where the CSD’s properties changed in unoperated females and in males that underwent an orchiectomy compared to male animals [157,158].

In the FHM2 subtype of migraine, the ATP1A2 gene carries mutations located on the 1q23.2 chromosome and encodes the alpha-2 subunit of Na^+^/K^+^ ATPase [35]. This pump is responsible for transporting Na^+^ out of the cell and K+ into the cell. Na^+^/K^+^ ATPase primarily is found on astrocytes in the nervous system and is involved in the regulation of neurotransmitter clearance from the synaptic cleft by removing its excess K^+^. Numerous and different types of mutations have been identified of the ATP1A2 gene in FHM2 patients, which result in loss-of-function of the encoded ATPase, causing increased K^+^ concentration in the synaptic cleft, due to which the glutamate uptake decreases, and neuronal excitability increases [159,160]. Transgenic mice have also been created to model this type of migraine, which carry either a knock-in loss-of-function missense mutation in heterozygous form (the homozygous form is lethal [161,162]), knock-out mutations also in heterozygous form [163], or mutations conditionally deleted [164]. Heterozygous mice do not show clinical symptoms of FHM2 [160,161], while episodic paralysis is observed in knock-out mice where the mutation is induced by programmed method [164]. Changes in the behavior of heterozygous mutant mice are more indicative of psychiatric diseases like anxiety, fear, depression, and increased immobility [161,162], and some of them showed sexual dimorphism, suggesting here again the role of sex hormones in the pathomechanism of migraine [162]. At the same time, the susceptibility to CSD is increased in all strains [161,162,163,164], which may be related to decreased clearance of K^+^ and glutamate by cortical astrocytes due to neuronal activation and the smaller density of GLT-1a glutamate transporters in the cortical presynaptic astrocytes [165]. Interestingly, endoplasmic reticulum retention and subsequent proteosomal degradation decreased the mutant type of Na^+^/K^+^ ATPase protein in brain lysates [161]. 

The FHM3 subtype of migraine is related to the SCN1A gene located on the 2q24.3 chromosome and its mutations. This gene encodes the α1 subunit of the voltage-dependent neuronal NaV1.1 sodium channel [36], which plays an important role in the generation and propagation of action potentials in cortical neurons and GABAergic inhibitory interneurons [166]. In migraineurs suffering from FHM3, some mainly missense mutations were identified as causing a gain of function of the NaV1.1 channel, with complex functional consequences [167,168]. Some transgenic mouse strains exist to model the FHM3 at a preclinical level [166,169,170]. The homozygous mice carrying a SCN1A-R1648H mutation exhibit spontaneous generalized seizures and premature death, while the heterozygous mice showed infrequent spontaneous generalized seizures and increased susceptibility for seizures induced by flurothyl and by hyperthermia, mimicking febrile seizures in which interneurons seem to play a major role leading to decreased inhibition [166]. The mutant mice expressing a SCN1A-L263V mutation demonstrated spontaneous CSDs that propagated from the visual to the motor cortex [169]. The third mutant mouse strain carries the mutation of SCN1A-L1649Q, where homozygous mice died prematurely, whereas heterozygous mice had a normal lifespan [170]. Heterozygous mice displayed a significantly enhanced susceptibility to CSD, which is assumed to be due to interneuron hyperactivity [170].

In FHM4, the gene encoding PRRT2 protein is mutated. It is located on the 16p11.2 chromosome and participates in the regulation of neurotransmitter release, affects the function of several types of ion channels, and influences the synaptogenesis. Mutations affecting the gene cause the loss of the function of protein, and the decreased amount of protein is not sufficient for complete function, leading to abnormal signaling between neurons, affecting the activity of ion channels and modifying the synaptogenesis. To better investigate the PRRT2 mutation’s effect, it is possible to use homozygous PRRT2 knock-out mice. These animals are viable but show paroxysmal movements and responses with abnormal motor behaviors to audiogenic stimuli and are more sensitive to the convulsive effects of pentylenetetrazol [171]. Related to movement abnormalities, high-frequency stimulation induced higher excitatory strength at parallel fiber–Purkinje cell synapses in cerebellar slices, indicating specific effects in the cerebellum [171]. Analysis of synaptic function showed changes detected at the level of excitatory neurons and inhibitory neurons that result in a state of heightened spontaneous and evoke activity at the network level, suggesting the network’s instability or hyperexcitability, which can be a possible basis of the paroxysmal phenotypes associated with PRRT2 mutations [172]. During synaptogenesis in cultured hippocampal neurons, morphology of the growth cones is altered in neurons derived from PRRT2 KO mice, which is associated with a selective alteration of the actin–cytoskeleton dynamics, supporting a key role of this protein in the regulation of growth cone morphology during neuronal development [173].

**Figure 3:**
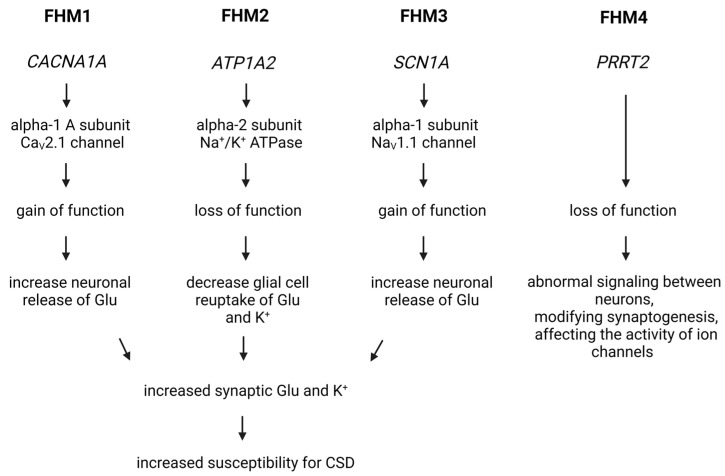
Genetic Insights into Familial Hemiplegic Migraine and Migraine Mechanisms. In the case of FHM1, a mutation in the CACNA1A gene increases the activity of the CaV2.1 voltage-gated calcium channel, facilitating the influx of calcium into cells and leading to increased glutamate release and heightened excitability of nerve cells. For FHM2 and FHM3, mutations affect the function of the Na^+^/K^+^ ATPase and the NaV1.1 sodium channel, influencing the transmission of nerve impulses. In FHM4, a mutation in the PRRT2 gene impacts neurotransmitter release and ion channel function, contributing to migraine symptoms. Cortical spreading depression is a key mechanism in triggering migraine headaches in these models. Glu, glutamate; CSD, cortical spreading depression.

Based on numerous studies, the overall conclusion is that FHM is one of the main models of migraine aura, in which cortical spreading depression as a result of increased cortical excitability triggers the migraine headache (Figure 3).

In addition to the mutations associated with FHM, there are also some other monogenic mutations that may be associated with migraine, such as KCNK18 mutations [173], CSNK1D mutations [174], and NOTCH3 mutations [175]. Results have been obtained in transgenic animal models of these mutations and suggest that they may indeed be associated with migraine, but this requires further research [156,174,176].

In conclusion, genetic models of migraine help us to understand certain aspects of the pathomechanism of migraine and can be a good tool for personalized medicine and biomarker and drug research. But it is not yet possible to create the perfect genetic migraine model with the knowledge we currently have because there is no consensus currently on the exact genes that clearly predispose to migraine, and it is problematic to create a polygenetic animal model. Unfortunately, it was also established by GWAS and PRS studies that genes associated with monogenic types of migraine probably do not play a stronger role in common migraine compared to other genes associated with migraine, increasing alone the overall risk for migraine only slightly [129]. However, in investigation of monogenic migraine, GWAS and PRS studies unanimously support the fact that the neurovascular unit plays a prominent role in migraine since a significant part of the proteins encoded by genes associated with migraine can be connected to the nervous or vascular systems.

## 4. Conclusions

Migraine, an extremely common primary headache type, has been a subject of scientific inquiry for a long time. Despite extensive research, the exact pathophysiological mechanism behind migraine remains elusive. However, in this field of investigation, animal experimental models have become of paramount importance, offering deeper insight into the triggering factors and biological foundations of migraines. In this article, we reviewed the significance of electrical and chemical stimulation models and delved into the mechanisms of migraine induced by nitroglycerin. Genetic modeling adds another layer to the picture, showcasing rare yet clinically important monogenic forms of migraine that help map out the genetic and biochemical foundations of the disease. The results from these models contribute not only to a better understanding of migraine pathophysiology but may also prove instrumental in identifying therapeutic targets and developing new treatment strategies.

## Data Availability

Not applicable.

## Abbreviations

5-HT_1B/D_ receptor	5-hydroxytryptamine 1B/1D receptor
CAMKIIα	Calcium/calmodulin-dependent protein kinase II alfa
CFA	Complete Freund’s adjuvant
cGMP	Cyclic guanosine monophosphate
CGRP	Calcitonin gene-related peptide
CM	Chronic migraine
CNS	Central nervous system
COX-2	Cyclooxygenase-2
CSD	Cortical spreading depression
EM	Episodic migraine
eNOS	Endothelial nitric oxide synthase
FHM	Familial hemiplegic migraine
GWAS	Genome-wide association studies
ICHD-3	The International Classification of Headache Disorders-3 beta
iNOS	Inducible nitric oxide synthase
IS	Inflammatory soup
KP	Kynurenine pathway
MOH	Medication-overuse headache
NF-κB	Nuclear factor kappa B
NI	Neurogenic inflammation
NMDA	N-methyl-D-aspartate
nNOS	Neuronal nitric oxide synthase
NO	Nitric oxide
NOS	Nitric oxide synthase
NSAIDs	Nonsteroidal anti-inflammatory drugs
NTG	Nitroglycerin
PACAP	Pituitary adenylate cyclase-activating polypeptide
PRS	Polygenic risk score
sGC	enzyme-soluble guanylyl cyclase
SSS	Superior sagittal sinus
TNC	Caudal trigeminal nucleus
TRPV1	Transient receptor potential vanilloid type 1
VIP	Vasoactive intestinal peptide
